# Activation of Heme Oxygenase Expression by Cobalt Protoporphyrin Treatment Prevents Pneumonic Plague Caused by Inhalation of *Yersinia pestis*

**DOI:** 10.1128/AAC.01819-19

**Published:** 2020-03-24

**Authors:** Joshua L. Willix, Jacob L. Stockton, Rachel M. Olson, Paul E. Anderson, Deborah M. Anderson

**Affiliations:** a Department of Veterinary Pathobiology and the Laboratory for Infectious Disease Research, University of Missouri, Columbia, Missouri, USA

**Keywords:** pneumonia, sepsis, heme oxygenase, cobalt protoporphyrin, *Yersinia pestis*, doxycycline, plague

## Abstract

Pneumonic plague, caused by the Gram-negative bacteria Yersinia pestis, is an invasive, rapidly progressing disease with poor survival rates. Following inhalation of Y. pestis, bacterial invasion of the lungs and a tissue-damaging inflammatory response allows vascular spread of the infection. Consequently, primary pneumonic plague is a multiorgan disease involving sepsis and necrosis of immune tissues and the liver, as well as bronchopneumonia and rampant bacterial growth. Given the likely role of the hyperinflammatory response in accelerating the destruction of tissue, in this work we evaluated the therapeutic potential of the inducible cytoprotective enzyme heme oxygenase 1 (HO-1) against primary pneumonic plague. On its own, the HO-1 inducer cobalt protoporphyrin IX (CoPP) provided mice protection from lethal challenge with Y. pestis CO92 with improved pulmonary bacterial clearance and a dampened inflammatory response compared to vehicle-treated mice. Furthermore, CoPP treatment combined with doxycycline strongly enhanced protection in a rat aerosol challenge model. Compared to doxycycline alone, CoPP treatment increased survival, with a 3-log decrease in median bacterial titer recovered from the lungs and the general absence of a systemic hyperinflammatory response. In contrast, treatment with the HO-1 inhibitor SnPP had no detectable impact on doxycycline efficacy. The combined data indicate that countering inflammatory toxicity by therapeutically inducing HO-1 is effective in reducing the rampant growth of Y. pestis and preventing pneumonic plague.

## Abstract (precis)

Pneumonic plague, caused by the Gram-negative bacteria Yersinia pestis, is an invasive, rapidly progressing disease with poor survival rates. Following inhalation of Y. pestis, bacterial invasion of the lungs and a tissue-damaging inflammatory response allows vascular spread of the infection. Consequently, primary pneumonic plague is a multiorgan disease involving sepsis and necrosis of immune tissues and the liver, as well as bronchopneumonia and rampant bacterial growth.

## INTRODUCTION

Plague, including the bubonic, pneumonic, and septicemic forms, is a lethal disease caused by Yersinia pestis. The incidence of plague has been reduced by modern public health infrastructure, but annual cases of the bubonic plague occur throughout the world due to stable sylvatic cycles of transmission between rodent and flea hosts (1). Recently, pneumonic plague was recognized as a reemerging disease in Madagascar, and in 2017 an outbreak of pneumonic plague accounted for 77% of the 2,417 cases with 209 deaths (2). Multiple independent antibiotic resistance mechanisms have emerged from the enzootic cycle, and it is thought that genetic engineering of Y. pestis could generate a drug-resistant weapon of mass destruction (3–6). For these reasons, there remains a need to develop nonantibiotic strategies for treatment of plague that are broadly protective.

Inhalation of Y. pestis by a mammalian host leads to the rapid development of acute bronchopneumonia and secondary sepsis that cause a high rate of mortality even when antibiotics known to be effective against bacterial growth are administered (1). In the lungs, Y. pestis initially suppresses inflammatory responses using two major thermally controlled virulence strategies: (i) the downregulation of *lpxM*, which causes hypoacylation of lipid A to a form that is less stimulatory to host cells, and (ii) the upregulation of the type III secretion system allowing the delivery of immune modulators into host cells (7–10). These factors are thought to provide immune evasion necessary for bacterial growth and the establishment of disease. Although the tetra-acylated lipid A effectively neutralizes host Toll-like receptor 4 and reduces the strength of the initial immune response, inflammatory toxicity occurs during the disease phase and accelerates the progression to lethality (11, 12). Dissemination of Y. pestis through the vasculature causes bacteremia and sepsis, allowing for the colonization of secondary tissues, including the liver and spleen (13–17). Late-stage disease is associated with inflammatory lesions, hemorrhage, and necrosis in these tissues, as well as in the lungs.

Protection from inflammatory damage has been demonstrated in multiple experimental models through the therapeutic induction of heme oxygenase (HO-1) by treatment with cobalt protoporphyrin IX (CoPP) (18–22). Heme oxygenase is an intracellular enzyme that degrades heme into Fe^2+^, carbon monoxide (CO), and biliverdin. During infection, heme exacerbates inflammation and oxidative tissue damage. Degradation of heme by HO-1 reduces these tissue-damaging responses, while the end products, CO and biliverdin, stimulate anti-inflammatory cytokine production and tissue repair (21, 23). The role of HO-1 in plague has not previously been reported.

Although many cell types and tissues are capable of inducing HO-1 expression, transcription is normally repressed and regulated by two independent mechanisms. When heme, or another metalloprotoporphyrin such as CoPP, is taken up by cells it binds to and inactivates the transcriptional repressor BTB domain and CNC homolog 1 (Bach1) (24). Loss of Bach1 binding allows transcriptional activators, primarily Nuclear factor (erythroid derived 2)-like 2 (Nrf2), access to bind the *Hmox1* promoter and activate expression. Nrf2 mediates a protective response to oxidative stress and not only activates *Hmox1* but also regulates other promoters, sometimes as a repressor, including proinflammatory cytokines such as interleukin-6 (IL-6) and tumor necrosis factor alpha (TNF-α) (25). The activity of Nrf2 is controlled by its sequestration and degradation in the cytoplasm due to its binding to Kelch-like ECH-associated protein 1 (Keap1) (26). Oxidative stress prevents this interaction, allowing Nrf2 to migrate into the nucleus to bind DNA. Although metalloprotoporphyrins all bind to Bach1 and induce *Hmox1* expression, they do not bind O_2_, so they are not degraded by HO-1, and most, except for CoPP, are competitive inhibitors of HO-1 that shut down its activity (27, 28). Metalloprotoporphyrins can therefore be used to therapeutically induce (CoPP) or inhibit (tin protoporphyrin IX or SnPP) HO-1 in experimental disease models. Here, we evaluated the therapeutic potential of HO-1 by using CoPP as a treatment for pneumonic plague.

## RESULTS

### Protection of mice from lethal intranasal challenge with *Y. pestis* by treatment with CoPP.

**FIG 1:**
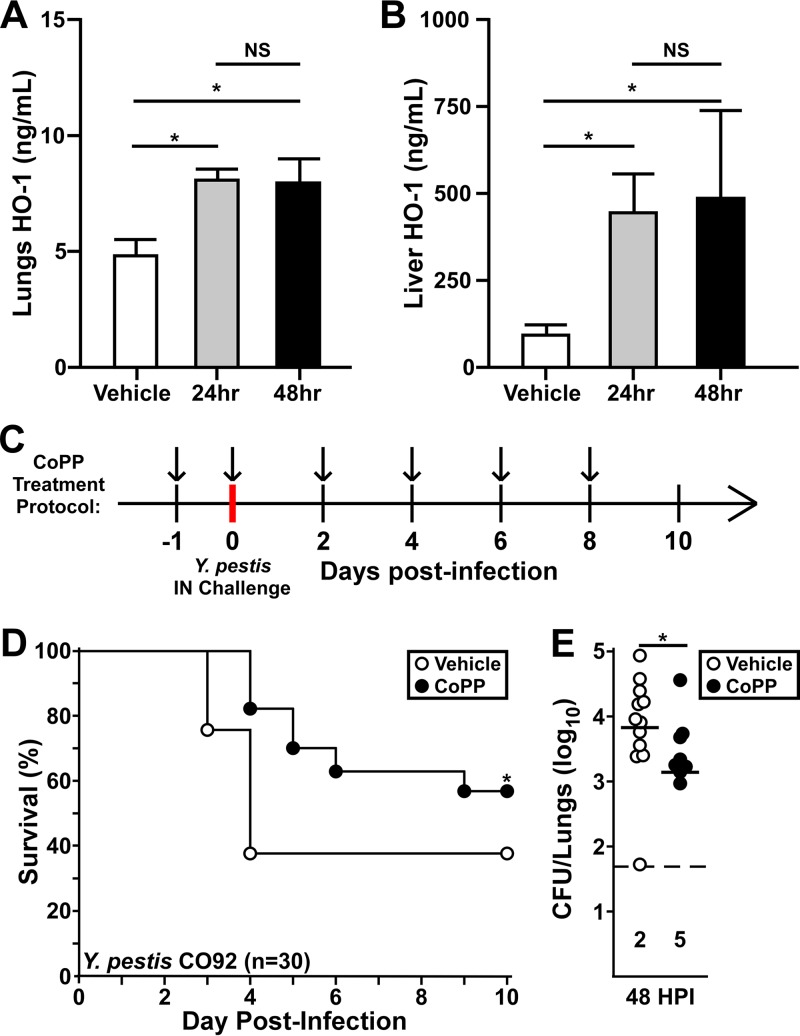
CoPP is protective against pneumonic plague in mice. (A and B) Groups of five mice (male and female) were provided CoPP treatment by intraperitoneal injection. Comparison between daily (24 h) and every-other-day (48 h) dosing was assessed by measuring HO-1 in the lung (A) and liver (B) homogenates 24 h after the second treatment. The data shown were collected in two independent trials (*n* = 10 per group). The data represent means with error bars showing standard deviations; statistical significance was evaluated by one-way ANOVA, followed by Dunnett’s multiple-comparison test (*, *P* < 0.05). (C) Dosing protocol for the mouse studies: i.p. treatment with CoPP (5 mg/kg, black arrows) or PBS vehicle was provided 24 h prior to challenge and on the day of challenge. Additional treatments were provided every other day for the duration of the 10-day observation period. (D and E) Groups of 10 C57BL/6 mice were treated with vehicle or CoPP following the protocol used for panel C and then challenged by intranasal infection with 5,000 to 8,000 CFU Y. pestis CO92. (D) Mice were monitored for survival for 10 days. The data shown were combined from three independent trials (*n* = 30 per group); the data were evaluated by a Gehan-Breslow log rank test (*, *P* < 0.05). (E) Groups of three to five male and female C57BL/6 mice, challenged and treated as shown for panel C, were euthanized at 48 h postinfection (hpi), and the bacterial titer was determined by serial dilution and plating. The dashed line represents the limit of detection, and the numbers below the line indicate the numbers of mice with undetectable bacteria. The data shown were collected in three independent trials (*n* = 13 per group); bars indicate the median. The data were evaluated by the Mann-Whitney test (*, *P* < 0.05).

Previous work in a murine model of LPS-induced inflammation showed that treatment of mice with CoPP 24 h prior to challenge was protective (20). To develop a treatment regimen for the plague model, we delivered CoPP by intraperitoneal (i.p.) injection to mice every 24 or 48 h and then measured HO-1 in the lungs and liver by ELISA. Compared to vehicle-treated mice, both treatment protocols resulted in significantly increased levels of HO-1 in the lungs and liver by 24 h posttreatment (Fig. 1A and B). To ensure sustained HO-1 expression throughout the Y. pestis study, we gave two treatments prior to challenge and then every 48 h postchallenge (Fig. 1C). Upon intranasal challenge with Y. pestis CO92, CoPP-treatment improved survival and increased the time to mortality, and by day 9 all surviving mice showed no sign of disease (Fig. 1D). We quantified Y. pestis in the lungs at 48 h postinfection (hpi) and found that CoPP treatment resulted in a significant reduction in bacterial burden, and half of the mice had no detectable bacteria (Fig. 1E).

**FIG 2:**
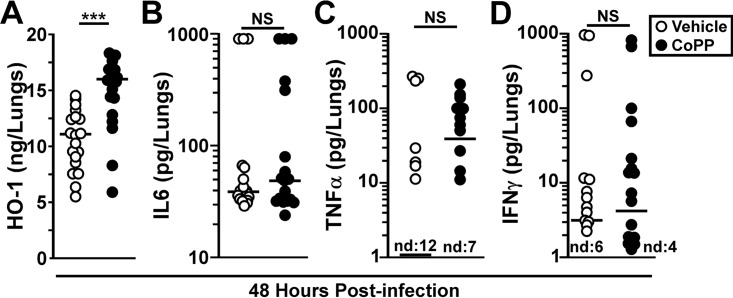
CoPP treatment does not induce inflammatory cytokines in the lungs during Y. pestis infection. Groups of five male and female C57BL/6 mice were treated as described for Fig. 1 with vehicle (open circles) or CoPP (closed circles) and challenged with Y. pestis CO92. At 48 hpi, the lungs were removed, homogenized in PBS, and analyzed for HO-1 (A), IL-6 (B), TNF-α (C), and IFN-γ (D) by ELISA. The data shown are combined from four independent trials (*n* = 20 per group). The data were analyzed by one-way ANOVA with Tukey’s posttest (*, *P* < 0.05; ***, *P* < 0.001). The bars indicate the median. nd, not detected.

When we probed for HO-1 in the lungs at 48 hpi, we found that it was increased in the CoPP treatment group compared to vehicle (Fig. 2A). Since CoPP treatment and HO-1 activity are expected to reduce inflammatory cytokine production, we measured lung IL-6 and TNF-α levels at 48 hpi. In both treatment groups, there were mice with elevated levels of IL-6, and the difference between the groups was not significant (Fig. 2B). The median concentration of pulmonary TNF-α was >10-fold higher in the CoPP treatment group, suggesting a possible correlation between TNF-α and reduced bacterial growth (Fig. 2C). We also measured gamma interferon (IFN-γ) since reduced IFN-γ has been associated with Y. pestis virulence (29). No differences in pulmonary IFN-γ levels were found between groups (Fig. 2D). Together, these data suggest that CoPP treatment may not substantially alter proinflammatory cytokine production and may work by changing the impact of the inflammatory response.

**FIG 3:**
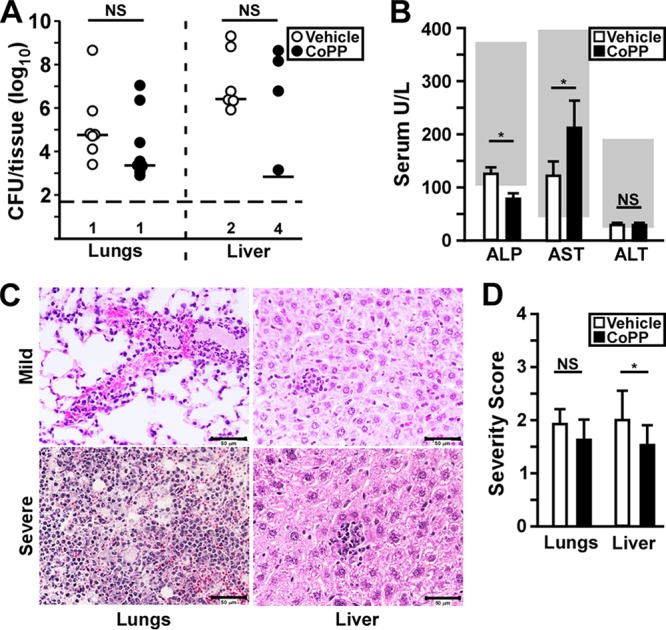
Loss of protection is associated with increased bacterial growth and liver injury. Groups of three to six C57BL/6 mice were treated as described for Fig. 1 with CoPP or vehicle, followed by intranasal infection with Y. pestis CO92. (A) Bacterial titer in the lungs and liver at 72 hpi. The bars indicate the median, and the horizontal dashed line represents the limit of detection. The numbers of mice with undetectable bacteria are indicated beneath (*n* = 10 per group, collected in two independent trials). (B) Liver function enzymes alkaline phosphatase (ALP), aspartate amino transferase (AST), and alanine amino transferase (ALT) were measured in the blood at 72 hpi. The data shown were collected in four independent trials (*n* = 20 to 22 per group) and depict the means of all the trials with the standard deviations. The gray boxes indicate the normal range for C57BL/6 mice. (C and D) Formalin-fixed lungs and liver samples were prepared at 72 hpi and processed for histopathology. (C) Representative images from CoPP-treated mice. (Left side, lungs) The upper panel represents mild to moderate inflammatory pathology found in most animals in this group, and the lower panel is the severe lesion found in one animal in this group showing neutrophil congestion, alveolar necrosis, and bacterial growth typical of primary pneumonic plague. (Right side, liver) Representative images found in the CoPP treatment group showing mild inflammatory lesions. (D) Mean severity score of lung pathology. The bars indicate the standard deviations. The data shown were collected in two independent trials and were analyzed for statistical significance by the Mann-Whitney (A) or unpaired *t* test (B and D) comparing treated to untreated (*, *P* < 0.05).

Since a number of mice succumbed in the CoPP treatment group, we sought to understand loss of protection by looking at the infection 24 h later. In contrast to 48 hpi, there was a large range of bacterial load recovered from the lungs of mice in both groups, and while the median titer was lower in the CoPP treatment group, this difference was not significant (Fig. 3A). In the liver 70% of the CoPP-treated mice had undetectable bacteria, and yet when bacteria were present, there appeared to be no direct impact of CoPP treatment on growth in the liver. We measured liver function enzymes in the blood and found significantly reduced alkaline phosphatase (ALP) and albumin between treatment groups, and in fact, the levels of ALP in the CoPP treatment group were below the normal range (Fig. 3B). Since hepatocytes are the major producer of ALP, this result may indicate reduced hepatocyte function in the CoPP treatment group. Aspartate aminotransferase, gamma globulin, total protein, and cholesterol were increased in the CoPP treatment group compared to vehicle though they remained within normal range, consistent with a moderate reduction in hepatocyte function (Fig. 3B; see Fig. S1A to D in the supplemental material). These results suggest that even though there was reduced bacterial growth in the liver in the CoPP treatment group, the disease in the liver may have been worsened by the treatment.

We therefore quantified pathological lesions in formalin fixed tissues collected at 72 hpi, scoring the severity of inflammatory and necrotic lesions in the lungs and liver. In the lungs, the lesion severity in both groups was moderate, with some containing neutrophil congestion, alveolar necrosis, hemorrhage and bacteria (Fig. 3C and D). This suggests that the frequency of pneumonia was relatively low in these mice, consistent with the low frequency of mice in both groups that harbored high bacterial load. We scored hepatocyte necrosis and inflammatory foci in the liver and found a small, but significant reduction in lesion severity in the CoPP treatment group. These data correlate with the reduction we observed in the colonization of the liver in the CoPP-treated mice at 72 hpi and suggest that CoPP did not cause an increase in hepatocyte necrosis.

**FIG 4:**
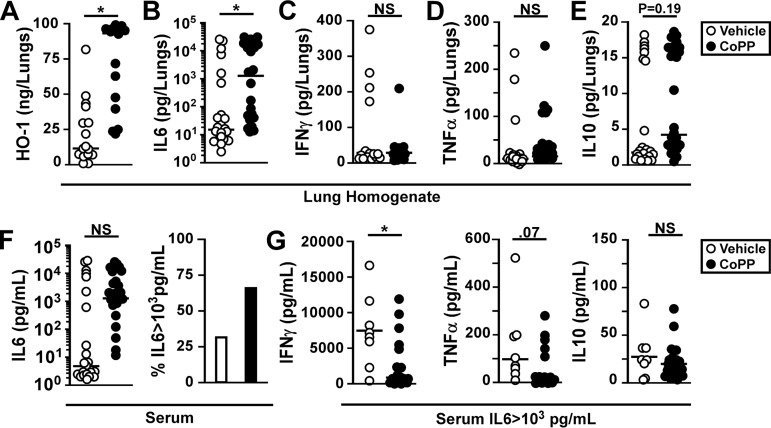
Loss of protection in CoPP-treated mice is associated with increased IL-6. Cytokine analysis of samples collected at 72 hpi (shown in Fig. 3). Filtered lung homogenates (A to E) and serum (F to G) were analyzed for the following proteins by ELISA or multiplex assay: HO-1 (A), IL-6 (B), IFN-γ (C), TNF-α (D), IL-10 (E), and serum IL-6 (F). The right panel indicates the percentage of mice with high levels of serum IL-6 (>1,000 pg/ml). (G) IFN-γ, TNF-α, and IL-10 from mice with high serum IL-6. Bars indicate the median. The data shown were collected in two to four independent trials and were analyzed for statistical significance by a Mann-Whitney test comparing treated to untreated (*, *P* < 0.05).

To verify that loss of protection did not correlate with an unexpected reduction in HO-1, we measured HO-1 in the lung homogenate at 72 hpi and found high levels of pulmonary HO-1 in the CoPP treatment group (Fig. 4A). In addition, the vehicle treatment group appeared to have induced HO-1 expression, with >10 ng/ml in the lung homogenates compared to <10 ng/ml observed in mice that were not infected (shown in Fig. 1A). Given these high levels of HO-1, we anticipated an increase in IL-10 and decrease in IL-6 in the lungs due to increased HO-1 activity. However, increased pulmonary IL-6 was present in the CoPP-treated mice (Fig. 4B). The levels of other cytokines in the lungs, including IFN-γ, TNF-α, and IL-10, were not significantly different between groups (Fig. 4C to E). These data suggest that the infection may alter the activity of or response to HO-1 such that there was little to no anti-inflammatory effect at 72 hpi.

In this experimental model (i.e., a low-volume intranasal challenge), untreated mice typically experience sepsis at 72 hpi, and we sought to determine whether this response was dampened or worsened by CoPP treatment (30). In the serum, we found that the median IL-6 titer in the CoPP treatment group was 1,000-fold higher than the vehicle-treatment group with ∼2-fold more animals in the CoPP treatment group having high levels of IL-6 (>1,000 pg/ml) and other cytokines, including IFN-γ and TNF-α but not IL-10, suggesting the onset of sepsis (Fig. 4F; see Fig. S2 in the supplemental material). We compared mice in both groups that harbored high serum IL-6 levels and found an apparent dampening of the hyperinflammatory response, such that IFN-γ and TNF-α levels in the CoPP treatment group appeared to be lower than in the vehicle group (Fig. 4G). The combined data may indicate that protection was limited to the primary lung infection.

### CoPP treatment enhances antibiotic efficacy in the rat pneumonic plague model.

Mice challenged by low-volume intranasal infection with Y. pestis CO92 have significant amounts of challenge material retained in the upper respiratory tract, where dissemination and secondary disease develop without primary lung involvement (30, 31). To test the efficacy of CoPP treatment on primary pneumonic plague in a relevant animal model, we evaluated aerosol challenge of rats in an antibiotic treatment model. Rats that are challenged by aerosolized Y. pestis rapidly develop primary lung infection and succumb to disease with severe bronchopneumonia (30, 32). In this study, we chose to use the outbred strain Sprague-Dawley (SD) rats (*Rattus rattus*) as the test subjects since this strain has been previously well characterized in its response to CoPP treatment and is known to be sensitive to Y. pestis infection (33–39). To determine a dose where there is at least 90% lethality, we conducted a dose escalation experiment in a pilot study. In female rats, we observed 100% lethality at a mean presented dose of 5.5 × 10^4^ CFU (Fig. S3A), whereas the male rats in the same cohort received over 9.5 × 10^4^ CFU but survived, suggesting they may be more resistant to infection than females. When we increased the challenge dose to 2.3 × 10^5^ CFU, 100% lethality was observed in the males (Fig. S3B). Disease progression was very rapid in male and female SD rats, causing lethality by days 2 and 3 postinfection. Because of the small difference in susceptibility, we challenged male and female rats separately in the treatment studies, targeting presented doses where 100% lethality was expected in control animals.

**FIG 5:**
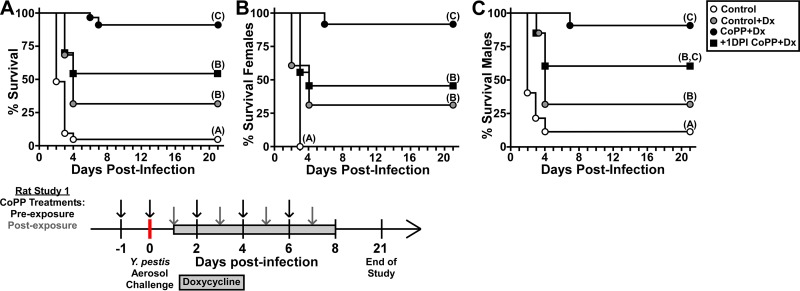
CoPP treatment enhances antibiotic efficacy against primary pneumonic plague in the rat model. Male and female rats (cohorts of 20 to 21 animals, with 5 to 6 rats/treatment group) were challenged by aerosolized Y. pestis CO92 (individual animal doses are shown in Table S3 in the supplemental material). Oral doxycycline treatment was given daily, beginning 24 h postchallenge (Control+Dx). CoPP treatment was initiated 24 h prior to challenge (CoPP+Dx) or 24 h postchallenge (+1DPI CoPP+Dx). Control animals received PBS and water (Control). Treatments continued for 7 days. Animals were monitored for a 21-day observation period. (A) Combined data, males and females; (B) females only; (C) males only. The data shown were collected in a total of four independent trials (two trials for each sex). Data were evaluated by a Gehan-Breslow-Wilcoxon log rank test (*n* = 20 to 22 per group). Different letters in parentheses indicate statistical significance between groups.

Previous work established that twice daily doses of 40 mg/kg intragastric (i.g.) doxycycline can protect mice from aerosol challenge with Y. pestis (40). We therefore gave once daily doses of half strength (20 mg/kg) in the rat model beginning 24 hpi as a subprotective treatment. To test CoPP efficacy, we either began dosing 24 h prior to infection as we did in the mouse model or 1 day postinfection (+1 dpi). Control animals, male and female, that received water by oral gavage succumbed to disease on days 2 to 4, with only 5% surviving the infection (Fig. 5A). Rats treated with doxycycline showed a small improvement with 25% survival and lethality occurring 3 to 4 days postinfection. In contrast, preexposure CoPP treatment greatly enhanced the efficacy of doxycycline, with 95% survival of animals in this group. When CoPP began 24 h postinfection, there was no significant difference in survival of female rats compared to the doxycycline treatment group (Fig. 5B). However, there was a small improvement in efficacy in the male rats, with >50% survival (Fig. 5C). Overall this study demonstrates that treatment of rats with CoPP, strongly enhances doxycycline efficacy against primary pneumonic plague.

### Induction of HO-1 leads to improved pulmonary clearance of *Y. pestis* in the rat pneumonic plague model.

**FIG 6:**
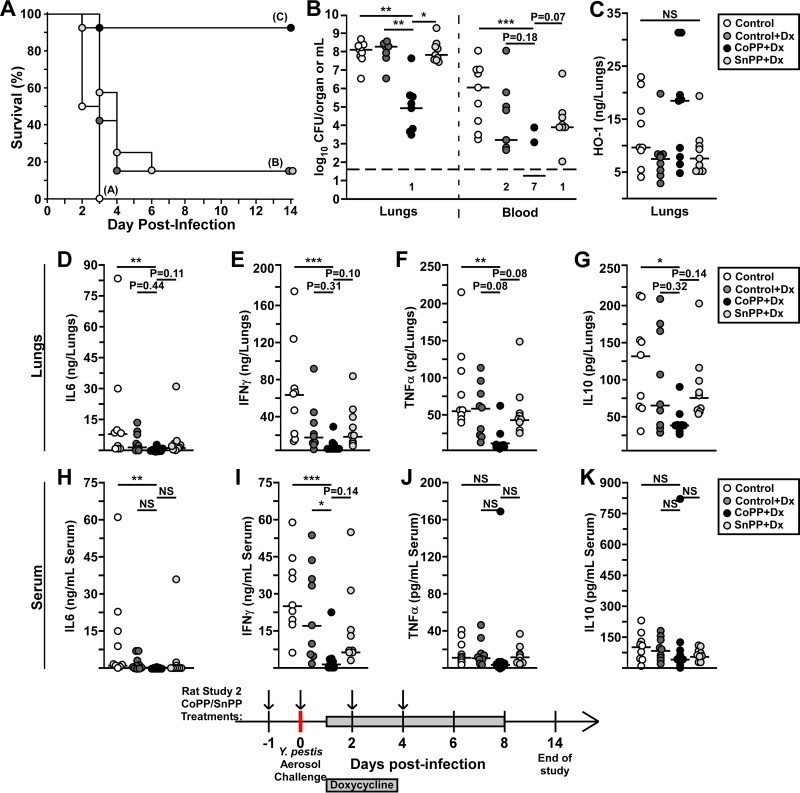
Stimulation of HO-1 enhances doxycycline efficacy in preventing bacterial growth. Groups of 28 (*n* = 7 per group) male SD rats were challenged by inhalation exposure to Y. pestis CO92 (individual doses shown in Table S3 in the supplemental material). For the study design, the rats were pretreated with 5 mg/kg CoPP (CoPP+Dx) or SnPP (SnPP+Dx) i.p. on days −1, 0, +2, and +4. Doxycycline (Dx) treatment (20 mg/kg IG) began 24 hpi and continued once daily for 7 days. Vehicle control animals received PBS by i.p. injection and water by oral gavage (control). (A) For each group, four rats were monitored for survival over 14 days. (B to L) The remaining three rats per group were euthanized at 48 hpi. The lungs (B to G) and blood (B, H to K) were removed and processed for CFU (B), HO-1 (C), IL-6 (D and H), IFN-γ (E and I), TNF-α (F and J), and IL-10 (G and K). The data shown were collected in three independent trials (*n* = 9 to 12 per group). The bars indicate median values. Data were pooled for statistical analyses by the Gehan-Breslow log rank (A) or Kruskal-Wallis test, followed by Dunn’s multiple-comparison test (B to K). *, *P* < 0.05; **, *P* < 0.01; ***, *P* < 0.001; NS, not significant. LH, lung homogenate.

Tin protoporphyrin IX (SnPP) is a potent inhibitor of HO-1 *in vitro* and *in vivo* with a long serum half-life and absorption in tissues, including the lungs and liver (33). To determine the effect of inhibiting HO-1, we compared efficacy of CoPP treatment to that of SnPP in male rats challenged by aerosol exposure to Y. pestis in the doxycycline treatment model. Strikingly, whereas CoPP treatment improved the efficacy of doxycycline, SnPP treatment had no effect (Fig. 6A). Rats that succumbed had developed primary pneumonic plague, typified by bacterial microcolonies, neutrophilic recruitment, alveolar necrosis, pulmonary edema, and hemorrhage (Fig. S4A to C). Furthermore, CoPP greatly enhanced pulmonary bacterial clearance, with a 3-log reduction in the median bacterial titer recovered from the lungs compared to all other groups (Fig. 6B). In addition, very few rats in the CoPP group had bacteremia, whereas 100% of the controls and about 80% of the doxycycline and SnPP groups harbored Y. pestis in the blood. These data indicate that the reduction of pulmonary Y. pestis delayed or prevented the development of secondary infection. In contrast, the inhibition of HO-1 by SnPP did not have a detectable impact on bacterial growth.

We also measured HO-1 and inflammatory cytokines in the lungs and serum at 48 hpi. Somewhat unexpectedly, there were no significant differences in HO-1 concentration in the lungs between any of the groups at 48 hpi, with considerable variation in the amounts within in each group (Fig. 6C). This may indicate that the infection induces HO-1. To determine whether the animals were experiencing a hyperinflammatory response, we measured pulmonary and serum cytokines. Pulmonary IL-6, IFN-γ, TNF-α, and IL-10 levels were significantly reduced in the CoPP group compared to vehicle (Fig. 6D to G). However, although there were overall lower levels of these cytokines in the CoPP group compared to doxycycline alone or SnPP, the differences were not significant, indicating a small effect conferred by doxycycline.

Serum cytokines, including IL-6, IFN-γ, TNF-α, and IL-10, were elevated in the control groups and in SnPP-treated rats indicating sepsis (Fig. 6H to K). In contrast, sepsis was not present in the CoPP-treated rats, with significantly lower serum IL-6 and IFN-γ (Fig. 6J and K). Overall, the combined data suggest that the CoPP and doxycycline combined for a synergistic improvement in controlling pulmonary bacterial growth and vascular spread, resulting in strong protection from primary pneumonic plague.

## DISCUSSION

Pneumonic plague is a deadly disease that consists of fulminant bronchopneumonia and severe sepsis. In this work, we showed that this can be prevented by treatment with the heme analog and inducer of HO-1 expression, cobalt protoporphyrin IX. On its own, CoPP treatment of mice appeared to reduce inflammatory toxicity, rather than suppress cytokine production during pulmonary Y. pestis infection. This allowed for improved bacterial clearance by the innate immune response. Synergistic protection with antibiotics was observed in a rat doxycycline treatment model. Since it is known that doxycycline efficacy is dependent on host neutrophils, these data suggest that HO-1 may improve the neutrophilic response to Y. pestis (40).

Previous work has established a role for HO-1 in improving the bactericidal mechanisms of neutrophils, and in decreasing damage to tissues caused by release of reactive oxygen species (ROS) by neutrophils (41, 42). In oxygen-rich environments, such as the lungs, free iron generated as a result of hemolysis leads to the generation of ROS that is proinflammatory and cytotoxic to cells (21). Highly virulent and invasive pathogens, such as Y. pestis, are likely able to exploit this response and grow, resulting in a feedback loop of neutrophilic inflammation and tissue damage that favors bacterial growth. Further protection from ROS may be provided by biliverdin and CO, produced by heme degradation, which have antiapoptotic and anti-inflammatory effects that could dampen immunopathology (22). Future work examining the activity of CoPP-treated macrophages or neutrophils *in vitro* and *in vivo* should be informative in understanding which, if any, of these mechanisms results in protection from pneumonic plague.

CoPP allows for Nrf2-regulated gene expression, an anti-oxidant program with pleiotropic effects, including an overall suppression of the inflammatory response (26). During Y. pestis infection of mice, however, this response was not observed, and in fact, increased IL-6 was found. This may be a consequence of modulation of host cell signaling by Y. pestis virulence factors or is an indication that essential costimulatory signals were not present. Without doxycycline, CoPP provided moderate protection and, in fact, it appeared that loss of protection may have been caused by off-target effects. For example, we found abnormally low ALP, elevated IL-6, and other modest changes in the serum of mice in the CoPP treatment group that may suggest liver toxicity. Other heme-binding proteins, primarily cytochrome P450 in the liver, are known to bind to and be inhibited by CoPP (43). Alternatively, overproduction of HO-1 in the liver may have unwanted effects (44). Additional investigation is needed to understand the mechanism underlying these observations, whether it is caused by HO-1 or CoPP directly, and whether reducing this effect improves protection. Nevertheless, the targeting of HO-1 or another cytoprotective mechanism to limit inflammatory damage is a promising treatment strategy for pneumonic plague.

Here, we observed an unexpected difference between male and female SD rats in their susceptibility to aerosol challenge with Y. pestis. In human plague, there are no known sex dependent differences in susceptibility, though historically, there have been more male than female plague victims, Sexual dimorphism has been frequently documented in infectious diseases, and in general, females show greater humoral and cell mediated responses than males of the same age and species, making females more resistant (45). In contrast, in the pneumonic plague model, SD rat females were more sensitive to infection by aerosolized Y. pestis, suggesting the effects we observed may result from the unique host-pathogen interactions that define plague. Since the disease course appeared to have the same kinetics and outcome in both sexes, we think it likely that the difference in susceptibility relates to an early event that impacts the initiation of infection. In an endotoxin challenge model, the inflammatory response of the SD rat female involved higher production of inflammatory cytokines from alveolar macrophages (46). Alveolar macrophages are early cellular targets of Y. pestis infection of the lungs, where such an effect could impact the initiation of infection (47). Future treatment and vaccine studies of plague should include male and female animals until there is a better understanding of the mechanism underlying sex-dependent susceptibility in the SD rat.

## MATERIALS AND METHODS

### Bacterial strains.

Yersinia pestis CO92 is a wild-type (Orientalis biovar) strain originally isolated from a plague patient (48). Bacteria were routinely grown from frozen stock by streaking for isolation onto heart infusion agar (HIA) plates (nonpigmented strains) or HIA plates supplemented with 0.005% (wt/vol) Congo red and 0.2% (wt/vol) galactose (for CO92) to screen bacteria that retain the pigmentation locus (49). For intranasal challenge studies, a single pigmented colony was used to inoculate heart infusion broth (HIB) supplemented with 2.5 mM CaCl_2_ and grown for 18 to 24 h at 37°C and 125 rpm. For aerosol challenge, bacteria were prepared as previously described (50). Briefly, Y. pestis CO92 seed stocks were used to inoculate HIB plus 2.5 mM CaCl_2_ and grown for 20 to 22 h at 37°C and 125 rpm. Bacteria were sedimented by centrifugation, followed by resuspension in 10 ml of sterile PBS. Presented dose was calculated using Guyton’s formula after verifying nebulizer and impinger concentrations by plating on HIA with Congo red (51). All work with the wild-type Y. pestis strain CO92 was performed in a select agent-authorized biosafety level 3 laboratory.

### Vertebrate animals.

**(i) Ethics statement.** All animal procedures were in compliance with the Office of Laboratory Animal Welfare and the National Institutes of Health *Guide for the Care and Use of Laboratory Animals* and were approved by the University of Missouri Animal Care and Use Committee.

**(ii) Mouse model.** Wild-type C57BL/6 mice were used for the mouse studies. Breeder pairs were purchased (Jackson Laboratories, Bar Harbor, ME), and mice were reared at the University of Missouri. Approximately equal numbers of male and female, aged-matched mice, ranging from 15 to 30 g were used. Cobalt protoporphyrin IX (CoPP; Enzo Life Sciences, New York, NY) was solubilized in 0.1 M NaOH and neutralized to pH 7 with HCl. Mice were given 5 mg/kg CoPP, or an equivalent volume of vehicle control, by intraperitoneal injection per treatment.

Mice were challenged with Y. pestis CO92 by intranasal instillation of a 10-μl volume as previously described (30). The actual CFU were verified by plating a serial dilution of the challenge material on HIA. Infected mice were monitored by daily health assessment for signs of progressing disease, including reduced activity, hunched posture, lack of grooming, and labored breathing, as well as other less common signs such as ocular discharge.

**(iii) Rat model.** Age-matched male and female Sprague Dawley rats (12 to 16 weeks old) were used for this study and were obtained from Charles River Laboratories. Rats were housed for >7 days in the ABSL3 facility prior to initiating the study. Approximately 2 × 10^9^ CFU/ml was placed in the sparging liquid aerosol generator nebulizer in a nose-only inhalation exposure system (CH Technologies, Westwood, NJ). The aerosol conditions were 65% humidity, with a 10- to 20-min exposure time. Two Teflon impingers were placed at the base of the exposure tower to measure Y. pestis concentration in the aerosol. In each trial, males and females were age matched and, within each cohort, weight differences were <10%. Age-matched male and female rats differed in weight by as much as 50%, resulting in significant differences in the volume of air (and challenge material) inhaled. Therefore, to control dosing, males and females were challenged in separate cohorts. Actual presented dose was calculated as aerosol concentration × minute volume (MV) × exposure time. For each trial, all animals were challenged in a single cohort. Doses of individual animals are shown in Tables S1 to 3 in the supplemental material.

Rats were given 5 mg/kg CoPP (or SnPP where indicated) by intraperitoneal injection per treatment. In different studies, the duration of CoPP treatment was progressively reduced to minimize the potential toxicity of the treatment without detectable impact on the survival outcome. Oral doxycycline (20 mg/kg Vibramycin) or water was given once daily by gavage beginning 24 h postexposure for 7 days. Rats were weighed daily and scored for the appearance of clinical signs of disease, including hunched posture, poor grooming, reduced activity, porphyrin discharge, and labored breathing.

All animals that survived to the end of the observation period (10, 14, or 21 days as indicated in the figures) or were identified as moribund (defined by pronounced neurologic signs, inactivity, and severe weakness) were euthanized by CO_2_ asphyxiation, followed by bilateral pneumothorax or cervical dislocation, methods approved by the American Veterinary Medical Association Guidelines on Euthanasia.

### CFU determination.

At the indicated time points postinfection, animals were euthanized, and tissues were aseptically removed and homogenized in sterile phosphate-buffered saline (PBS). Samples were serially diluted and plated on HIA in duplicate for the enumeration of viable bacteria. The limit of detection was 50 CFU/ml.

### Histopathology.

After euthanasia, lungs were perfused *in situ* with 10% formalin; the lungs, liver, and spleen were removed and incubated in 10% formalin for 2 days. Organs were then prepared for embedment, blocked in wax, and cut into 5-μm sections. Tissue sections were stained with hematoxylin and eosin, and stained slides were affixed with permanent coverslips. Sample identities were blinded for analysis. For quantification, inflammatory lesions, necrosis, hemorrhage, edema, and other lesions were scored for increasing severity in size and/or frequency in at least 10 nonoverlapping fields for each tissue. The appearance of bacterial colonies in the tissues was noted but not included in the lesion scoring if it was not causing tissue pathology.

### Protein quantification.

Blood, collected postmortem by cardiac puncture, and lungs, homogenized in sterile PBS, were centrifuged to remove cellular material. Serum and lung homogenate were antibiotic treated to inactivate Y. pestis and stored at –80°C until analysis. To quantify cytokines, serum and lung homogenate were analyzed by a multiplex cytokine assay (Millipore Sigma, St. Louis, MO) for proinflammatory cytokines known to play a role in plague: IFN-γ, TNF-α, IL-6, and IL-10. For some studies, an enzyme-linked immunosorbent assay (ELISA) was used: TNF-α, IL-6, and IFN-γ (R&D Systems, Minneapolis, MN) and HO-1 (Enzo Life Sciences, New York, NY). For some studies, aliquots of serum were sent for analysis of liver damage indicators (ALP, AST, and ALT), as well as total albumin, cholesterol, total protein, and total bilirubin (Comparative Clinical Pathology Services, LLC, Columbia, MO).

### Statistical evaluation.

Control conditions from all trials were compared by ANOVA to verify similarity in results and then combined for statistical evaluation. Statistical significance was assessed by the tests indicated in the figure legends using GraphPad software (GraphPad Software, La Jolla, CA). Significance was concluded when the *P* value was <0.05.

## Supplementary Material

Supplemental file 1
