# Supplementary material for: Activation of Heme Oxygenase Expression by Cobalt Protoporphyrin Treatment Prevents Pneumonic Plague Caused by Inhalation of Yersinia pestis
Source: Antimicrob Agents Chemother. 2020 Mar 24;64(4):e01819-19. doi: 10.1128/AAC.01819-19 (PMC7179272; doi:10.1128/AAC.01819-19)
Supplement: Supplemental file 1 [file AAC.01819-19-s0001.pdf]

Supplemental Information.

Table S1. Aerosol challenge dose for individual Sprague Dawley rats in pilot experiment (Figure S3).

Animal ID[a]	Weight (g)	MV[b](mL/min)	$Time_{exp}$ (min)	Neb $Conc_{meas}$ (CFU/mL)[c]	Aero $Conc_{meas}$ (CFU/mL)	Spray Factor	$Dose_{delivered}$ (CFU)
**Trial 1: males and females**							
1-01M	407	190.29	10	4.00E+09	48	1.20E-08	9.13E+04
1-02M	452	205.86	10	4.00E+09	48	1.20E-08	9.88E+04
1-03F	240	128.05	10	4.00E+09	48	1.20E-08	6.15E+04
1-04F	207	114.60	10	4.00E+09	48	1.20E-08	5.50E+04
**Trial 2: males and females**							
1-01F	253	133.22	15	1.68E+09	78.5	4.67E-08	1.57E+05
1-02F	264	137.54	15	1.68E+09	78.5	4.67E-08	1.62E+05
1-03F	263	137.15	15	1.68E+09	78.5	4.67E-08	1.61E+05
1-01M	438	201.06	15	1.68E+09	78.5	4.67E-08	2.37E+05
1-02M	430	198.30	15	1.68E+09	78.5	4.67E-08	2.33E+05
1-03M	418	194.13	15	1.68E+09	78.5	4.67E-08	2.29E+05

a: ID: identification; M:male, F:female

b: MV: minute volume

c: Nebulizer concentration of *Y. pestis* , CFU: colony forming units

Table S2. Aerosol challenge dose for individual Sprague Dawley rats for pre- and post-exposure efficacy study (Figure 5).

Animal ID	Weight (g)	MV (mL/min)	$Time_{exp}$ (min)	Neb $Conc_{meas}$ (CFU/mL)	Aero $Conc_{meas}$ (CFU/mL)	Spray Factor	$Dose_{delivered}$ (CFU)
**Trial 1: All male**							
1-1M	445	203.46	13	2.50E+09	100.0	4.00E-08	2.65E+05
1-2	487	217.70	13	2.50E+09	100.0	4.00E-08	2.83E+05
1-3	425	196.57	13	2.50E+09	100.0	4.00E-08	2.56E+05
1-4	414	192.74	13	2.50E+09	100.0	4.00E-08	2.51E+05
1-5	454	206.54	13	2.50E+09	100.0	4.00E-08	2.69E+05
1-6	426	196.91	13	2.50E+09	100.0	4.00E-08	2.56E+05
1-7	497	221.05	13	2.50E+09	100.0	4.00E-08	2.87E+05
1-8	534	233.28	13	2.50E+09	100.0	4.00E-08	3.03E+05
1-9	483	216.36	13	2.50E+09	100.0	4.00E-08	2.81E+05
1-10	447	204.15	13	2.50E+09	100.0	4.00E-08	2.65E+05
1-11	382	181.45	13	2.50E+09	100.0	4.00E-08	2.36E+05
1-12	487	217.70	13	2.50E+09	100.0	4.00E-08	2.83E+05
1-13	423	195.87	13	2.50E+09	100.0	4.00E-08	2.55E+05
1-14	468	211.30	13	2.50E+09	100.0	4.00E-08	2.75E+05
1-15	446	203.81	13	2.50E+09	100.0	4.00E-08	2.65E+05
1-16	395	186.07	13	2.50E+09	100.0	4.00E-08	2.42E+05
1-17	447	204.15	13	2.50E+09	100.0	4.00E-08	2.65E+05
1-18	498	221.38	13	2.50E+09	100.0	4.00E-08	2.88E+05
1-19	501	222.38	13	2.50E+09	100.0	4.00E-08	2.89E+05
1-20	372	177.88	13	2.50E+09	100.0	4.00E-08	2.31E+05
1-21	423	195.87	13	2.50E+09	100.0	4.00E-08	2.55E+05
**Trial 2: All female**							
1-1F	290	147.58	13	1.24E+09	50.4	4.06E-08	9.67E+04
1-2	279	143.36	13	1.24E+09	50.4	4.06E-08	9.39E+04
1-3	277	142.59	13	1.24E+09	50.4	4.06E-08	9.34E+04
1-4	292	148.34	13	1.24E+09	50.4	4.06E-08	9.72E+04
1-5	248	131.24	13	1.24E+09	50.4	4.06E-08	8.60E+04
1-6	286	146.05	13	1.24E+09	50.4	4.06E-08	9.57E+04
1-7	253	133.22	13	1.24E+09	50.4	4.06E-08	8.73E+04
1-8	260	135.97	13	1.24E+09	50.4	4.06E-08	8.91E+04
1-9	271	140.26	13	1.24E+09	50.4	4.06E-08	9.19E+04
1-10	250	132.03	13	1.24E+09	50.4	4.06E-08	8.65E+04
1-11	294	149.10	13	1.24E+09	50.4	4.06E-08	9.77E+04
1-12	248	131.24	13	1.24E+09	50.4	4.06E-08	8.60E+04
1-13	272	140.65	13	1.24E+09	50.4	4.06E-08	9.22E+04
1-14	257	134.79	13	1.24E+09	50.4	4.06E-08	8.83E+04

1-15	270	139.88	13	1.24E+09	50.4	4.06E-08	9.16E+04
1-16	280	143.74	13	1.24E+09	50.4	4.06E-08	9.42E+04
1-17	253	133.22	13	1.24E+09	50.4	4.06E-08	8.73E+04
1-18	240	128.05	13	1.24E+09	50.4	4.06E-08	8.39E+04
1-19	246	130.44	13	1.24E+09	50.4	4.06E-08	8.55E+04
1-20	262	136.76	13	1.24E+09	50.4	4.06E-08	8.96E+04
1-21	269	139.49	13	1.24E+09	50.4	4.06E-08	9.14E+04
**Trial 3: All male**							
2-1M	403	188.89	20	2.07E+09	82.8	4.00E-08	3.13E+05
2-2	379	180.38	20	2.07E+09	82.8	4.00E-08	2.99E+05
2-3	397	186.77	20	2.07E+09	82.8	4.00E-08	3.09E+05
2-4	397	186.77	20	2.07E+09	82.8	4.00E-08	3.09E+05
2-5	410	191.34	20	2.07E+09	82.8	4.00E-08	3.17E+05
2-6	387	183.23	20	2.07E+09	82.8	4.00E-08	3.03E+05
2-7	401	188.18	20	2.07E+09	82.8	4.00E-08	3.12E+05
2-8	384	182.17	20	2.07E+09	82.8	4.00E-08	3.02E+05
2-9	392	185.01	20	2.07E+09	82.8	4.00E-08	3.06E+05
2-10	398	187.12	20	2.07E+09	82.8	4.00E-08	3.10E+05
2-11	400	187.83	20	2.07E+09	82.8	4.00E-08	3.11E+05
2-12	368	176.44	20	2.07E+09	82.8	4.00E-08	2.92E+05
2-13	400	187.83	20	2.07E+09	82.8	4.00E-08	3.11E+05
2-14	399	187.48	20	2.07E+09	82.8	4.00E-08	3.10E+05
2-15	373	178.24	20	2.07E+09	82.8	4.00E-08	2.95E+05
2-16	386	182.88	20	2.07E+09	82.8	4.00E-08	3.03E+05
2-17	400	187.83	20	2.07E+09	82.8	4.00E-08	3.11E+05
2-18	407	190.29	20	2.07E+09	82.8	4.00E-08	3.15E+05
2-19	404	189.24	20	2.07E+09	82.8	4.00E-08	3.13E+05
2-20	370	177.16	20	2.07E+09	82.8	4.00E-08	2.93E+05
**Trial 4: All female**							
2-1F	237	126.85	12	2.45E+09	98.0	4.00E-08	1.49E+05
2-2	255	134.01	12	2.45E+09	98.0	4.00E-08	1.58E+05
2-3	232	124.83	12	2.45E+09	98.0	4.00E-08	1.47E+05
2-4	239	127.65	12	2.45E+09	98.0	4.00E-08	1.50E+05
2-5	260	135.97	12	2.45E+09	98.0	4.00E-08	1.60E+05
2-6	234	125.64	12	2.45E+09	98.0	4.00E-08	1.48E+05
2-7	248	131.24	12	2.45E+09	98.0	4.00E-08	1.54E+05
2-8	262	136.76	12	2.45E+09	98.0	4.00E-08	1.61E+05
2-9	243	129.25	12	2.45E+09	98.0	4.00E-08	1.52E+05
2-10	232	124.83	12	2.45E+09	98.0	4.00E-08	1.47E+05

2-11	224	121.59	12	2.45E+09	98.0	4.00E-08	1.43E+05
2-12	239	127.65	12	2.45E+09	98.0	4.00E-08	1.50E+05
2-13	232	124.83	12	2.45E+09	98.0	4.00E-08	1.47E+05
2-14	243	129.25	12	2.45E+09	98.0	4.00E-08	1.52E+05
2-15	243	129.25	12	2.45E+09	98.0	4.00E-08	1.52E+05
2-16	260	135.97	12	2.45E+09	98.0	4.00E-08	1.60E+05
2-17	268	139.10	12	2.45E+09	98.0	4.00E-08	1.64E+05
2-18	250	132.03	12	2.45E+09	98.0	4.00E-08	1.55E+05
2-19	257	134.79	12	2.45E+09	98.0	4.00E-08	1.59E+05
2-20	230	124.03	12	2.45E+09	98.0	4.00E-08	1.46E+05

Table S3. Aerosol challenge dose for individual Sprague Dawley rats for 48 hr/survival study (Figure 6).

Animal ID	Weight (g)	MV (mL/min)	$Time_{exp}$ (min)	Neb $Conc_{meas}$ (CFU/mL)	Aero $Conc_{meas}$ (CFU/mL)	Spray Factor	$Dose_{delivered}$ (CFU)
**Trial 1: All male**							
1-01	258	135.19	20	4.00E+09	83.8	2.10E-08	2.27E+05
1-02	286	146.05	20	4.00E+09	83.8	2.10E-08	2.45E+05
1-03	285	145.66	20	4.00E+09	83.8	2.10E-08	2.44E+05
1-04	287	146.43	20	4.00E+09	83.8	2.10E-08	2.45E+05
1-05	276	142.20	20	4.00E+09	83.8	2.10E-08	2.38E+05
1-06	287	146.43	20	4.00E+09	83.8	2.10E-08	2.45E+05
1-07	275	141.81	20	4.00E+09	83.8	2.10E-08	2.38E+05
1-08	266	138.32	20	4.00E+09	83.8	2.10E-08	2.32E+05
1-09	284	145.28	20	4.00E+09	83.8	2.10E-08	2.43E+05
1-10	269	139.49	20	4.00E+09	83.8	2.10E-08	2.34E+05
1-11	274	141.43	20	4.00E+09	83.8	2.10E-08	2.37E+05
1-12	278	142.97	20	4.00E+09	83.8	2.10E-08	2.40E+05
1-13	301	151.76	20	4.00E+09	83.8	2.10E-08	2.54E+05
1-14	292	148.34	20	4.00E+09	83.8	2.10E-08	2.49E+05
1-15	286	146.05	20	4.00E+09	83.8	2.10E-08	2.45E+05
1-16	276	142.20	20	4.00E+09	83.8	2.10E-08	2.38E+05
1-17	241	128.45	20	4.00E+09	83.8	2.10E-08	2.15E+05
1-18	268	139.10	20	4.00E+09	83.8	2.10E-08	2.33E+05
1-19	255	134.01	20	4.00E+09	83.8	2.10E-08	2.25E+05
1-20	250	132.03	20	4.00E+09	83.8	2.10E-08	2.21E+05
1-21	269	139.49	20	4.00E+09	83.8	2.10E-08	2.34E+05
1-22	248	131.24	20	4.00E+09	83.8	2.10E-08	2.20E+05
1-23	263	137.15	20	4.00E+09	83.8	2.10E-08	2.30E+05
1-24	274	141.43	20	4.00E+09	83.8	2.10E-08	2.37E+05
1-25	277	142.59	20	4.00E+09	83.8	2.10E-08	2.39E+05
1-26	253	133.22	20	4.00E+09	83.8	2.10E-08	2.23E+05
1-27	252	132.82	20	4.00E+09	83.8	2.10E-08	2.23E+05
1-28	272	140.65	20	4.00E+09	83.8	2.10E-08	2.36E+05
**Trial 2: All male**							
2-01	277	142.59	19	4.60E+09	1015.8	2.21E-07	2.75E+06

2-02	299	151.00	19	4.60E+09	1015.8	2.21E-07	2.91E+06
2-03	250	132.03	19	4.60E+09	1015.8	2.21E-07	2.55E+06
2-04	305	153.27	19	4.60E+09	1015.8	2.21E-07	2.96E+06
2-05	297	150.24	19	4.60E+09	1015.8	2.21E-07	2.90E+06
2-06	289	147.19	19	4.60E+09	1015.8	2.21E-07	2.84E+06
2-07	317	157.77	19	4.60E+09	1015.8	2.21E-07	3.04E+06
2-08	322	159.63	19	4.60E+09	1015.8	2.21E-07	3.08E+06
2-09	296	149.86	19	4.60E+09	1015.8	2.21E-07	2.89E+06
2-10	288	146.81	19	4.60E+09	1015.8	2.21E-07	2.83E+06
2-11	263	137.15	19	4.60E+09	1015.8	2.21E-07	2.65E+06
2-12	278	142.97	19	4.60E+09	1015.8	2.21E-07	2.76E+06
2-13	275	141.81	19	4.60E+09	1015.8	2.21E-07	2.74E+06
2-14	284	145.28	19	4.60E+09	1015.8	2.21E-07	2.80E+06
2-15	338	165.54	19	4.60E+09	1015.8	2.21E-07	3.19E+06
2-16	291	147.96	19	4.60E+09	1015.8	2.21E-07	2.86E+06
2-17	272	148.72	19	4.60E+09	1015.8	2.21E-07	2.87E+06
2-18	229	149.10	19	4.60E+09	1015.8	2.21E-07	2.88E+06
2-19	285	142.59	19	4.60E+09	1015.8	2.21E-07	2.75E+06
2-20	278	155.52	19	4.60E+09	1015.8	2.21E-07	3.00E+06
2-21	317	152.89	19	4.60E+09	1015.8	2.21E-07	2.95E+06
2-22	269	141.81	19	4.60E+09	1015.8	2.21E-07	2.74E+06
2-23	265	152.13	19	4.60E+09	1015.8	2.21E-07	2.94E+06
2-24	294	154.02	19	4.60E+09	1015.8	2.21E-07	2.97E+06
2-25	273	138.32	19	4.60E+09	1015.8	2.21E-07	2.67E+06
2-26	288	147.58	19	4.60E+09	1015.8	2.21E-07	2.85E+06
2-27	287	155.90	19	4.60E+09	1015.8	2.21E-07	3.01E+06
2-28	303	138.71	19	4.60E+09	1015.8	2.21E-07	2.68E+06
**Trial 3: All male**							
3-01	269	139.49	20	3.80E+09	361.6	9.52E-08	1.01E+06
3-02	255	134.01	20	3.80E+09	361.6	9.52E-08	9.69E+05
3-03	282	144.51	20	3.80E+09	361.6	9.52E-08	1.05E+06
3-04	280	143.74	20	3.80E+09	361.6	9.52E-08	1.04E+06
3-05	330	162.59	20	3.80E+09	361.6	9.52E-08	1.18E+06
3-06	303	152.51	20	3.80E+09	361.6	9.52E-08	1.10E+06

3-07	291	147.96	20	3.80E+09	361.6	9.52E-08	1.07E+06
3-08	293	148.72	20	3.80E+09	361.6	9.52E-08	1.08E+06
3-09	269	139.49	20	3.80E+09	361.6	9.52E-08	1.01E+06
3-10	340	166.28	20	3.80E+09	361.6	9.52E-08	1.20E+06
3-11	301	151.76	20	3.80E+09	361.6	9.52E-08	1.10E+06
3-12	273	141.04	20	3.80E+09	361.6	9.52E-08	1.02E+06
3-13	288	146.81	20	3.80E+09	361.6	9.52E-08	1.06E+06
3-14	320	158.88	20	3.80E+09	361.6	9.52E-08	1.15E+06
3-15	269	139.49	20	3.80E+09	361.6	9.52E-08	1.01E+06
3-16	304	152.89	20	3.80E+09	361.6	9.52E-08	1.11E+06
3-17	293	148.72	20	3.80E+09	361.6	9.52E-08	1.08E+06
3-18	294	149.10	20	3.80E+09	361.6	9.52E-08	1.08E+06
3-19	277	142.59	20	3.80E+09	361.6	9.52E-08	1.03E+06
3-20	311	155.52	20	3.80E+09	361.6	9.52E-08	1.12E+06
3-21	304	152.89	20	3.80E+09	361.6	9.52E-08	1.11E+06
3-22	275	141.81	20	3.80E+09	361.6	9.52E-08	1.03E+06
3-23	302	152.13	20	3.80E+09	361.6	9.52E-08	1.10E+06
3-24	307	154.02	20	3.80E+09	361.6	9.52E-08	1.11E+06
3-25	266	138.32	20	3.80E+09	361.6	9.52E-08	1.00E+06
3-26	290	147.58	20	3.80E+09	361.6	9.52E-08	1.07E+06
3-27	312	155.90	20	3.80E+09	361.6	9.52E-08	1.13E+06
3-28	267	138.71	20	3.80E+09	361.6	9.52E-08	1.00E+06

Supplemental Information.

Figure S1

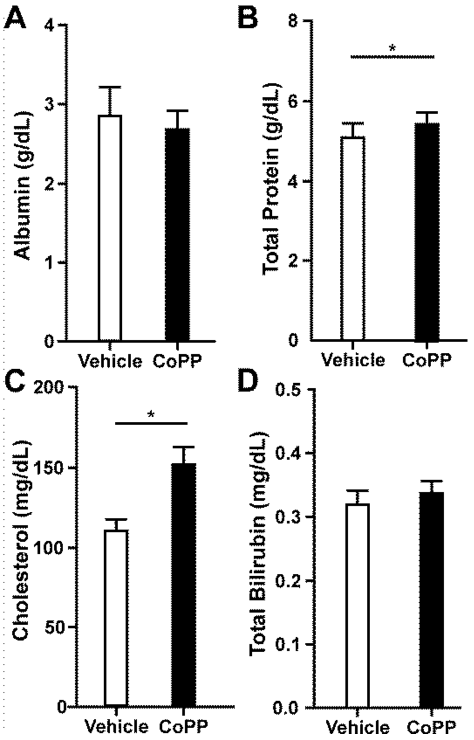

Supplemental Figure S1. *Liver function enzymes suggest negative impact of CoPP on the liver during Y. pestis infection.* Liver function tests were conducted on serum collected from vehicle and CoPP-treated mice at 72 HPI. (A) Albumin, (B) total protein, (C) cholesterol, and (D) bilirubin are shown (the remaining enzymes in the Liver Panel, ALP, ALT and AST, were shown in Figure 4). Data shown depict the mean and standard error of the mean. Data were collected in 4 independent trials, n=20 (vehicle) or 22 (CoPP), and analyzed by Student's t-test, *P<0.05.

Figure S2.

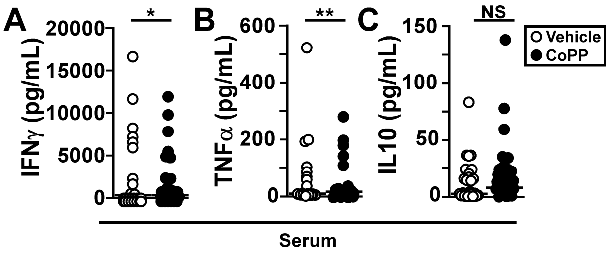

Supplemental Figure S2. *Loss of protection in CoPP-treated mice is associated with increased serum cytokines.* Cytokine analysis of serum samples collected at 72 HPI (remainder shown in figure 4). Serum was analyzed for the following proteins by Multiplex: (A) IFNγ; (B) TNFα; (C) IL10. Bars indicate median. Data shown were collected in 2-4 independent trials, and were analyzed for statistical significance by Mann Whitney test comparing treated to untreated, *P<0.05.

Figure S3.

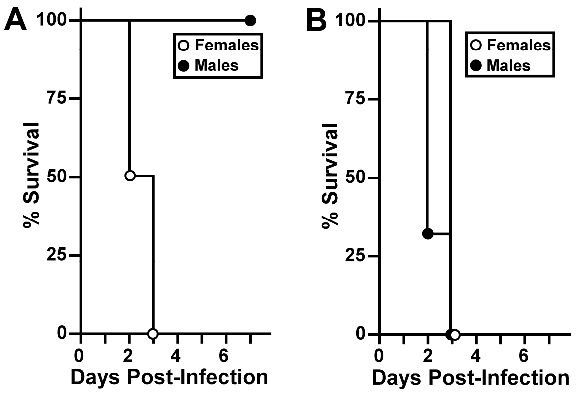

Supplemental Figure S3. *Challenge dose determination for Sprague Dawley rats*. A pilot challenge study was conducted to determine dosing. *Y. pestis* CO92 was prepared for aerosolization as described in the methods and delivered to groups of 2-3 age-matched female (open circles, weighing 210-265g) and male (closed circles, weighing 410-450g) Sprague Dawley rats by inhalation exposure. In cohort 1 (A), females received a mean presented dose of $5.5x10^4$ CFU and males received a mean dose of $9.5x10^4$ CFU; in cohort 2 (B), females received $1.6x10^5$ CFU, males received $2.3x10^5$ CFU. Individual animal doses shown in Supplemental Table S1. Survival was monitored over a 7 day observation period.

Figure S4.

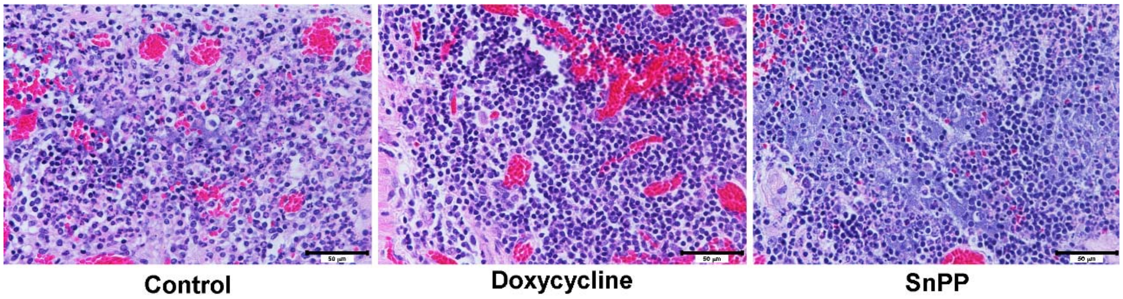

Supplemental Figure S4. *Post-mortem analysis of male rats indicates severe pneumonic disease.* Groups of 28 (n=7 per group) male Sprague-Dawley rats were challenged by inhalation exposure to *Y. pestis* CO92. Study design: Rats were pre-treated with 5mg/kg CoPP (CoPP+Dx) or SnPP (SnPP+Dx) IP on days -1, 0, +2, and +4; doxycycline (Dx) treatment (20mg/kg IG) began 24 HPI and continued once daily for 7 days. Vehicle control animals received PBS by IP injection and water by oral gavage (control). Animals that succumbed to disease were necropsied and the lungs inflated with 10% formalin, sectioned, then processed for hematoxylin and eosin (H&E) staining: Control group (left), Dx (center), or Doxycycline+SnPP treatment (right). Scale bar indicates 50µm. Data shown were collected from one trial.
